# Perceptions and Experiences of Research Participants on Gender-Based Violence Community Based Survey: Implications for Ethical Guidelines

**DOI:** 10.1371/journal.pone.0035495

**Published:** 2012-04-27

**Authors:** Yandisa Sikweyiya, Rachel Jewkes

**Affiliations:** 1 Gender and Health Research Unit, South African Medical Research Council, Pretoria, South Africa; 2 School of Public Health, University of the Witwatersrand, Johannesburg, South Africa; Research and Development Corporation, United States of America

## Abstract

**Objective:**

To explore how survey respondents perceived their experiences and the impact of participating in a survey, and to assess adverse consequences resulting from participation.

**Design:**

Qualitative study involving purposefully selected participants who had participated in a household-based survey.

**Methods:**

This qualitative study was nested within a survey that investigated the prevalence of gender-based violence perpetration and victimization with adult men and women in South Africa. 13 male- and 10 female-in-depth interviews were conducted with survey respondents.

**Results:**

A majority of informants, without gender-differences, perceived the survey interview as a rare opportunity to share their adverse and or personal experiences in a 'safe' space. Gender-differences were noted in reporting perceptions of risks involved with survey participation. Some women remained fearful after completing the survey, that should breach of confidentiality or full survey content disclosure occur, they may be victimized by partners as a punishment for survey participation without men's approval. A number of informants generally discussed their survey participation with others. However, among women with interpersonal violence history or currently in abusive relationships, full survey content disclosure was done with fear; the partner responses were negative, and few women reported receiving threatening remarks but none reported being assaulted. In contrast no man reported adverse reaction by others. Informants with major life adversities reported that the survey had made them to relive the experiences causing them sadness and pain at the time. No informant perceived the survey as emotionally harmful or needed professional support because of survey questions. Rather the vast majority perceived benefit from survey participation.

**Conclusion:**

Whilst no informant felt answering the survey questions had caused them emotional or physical harm, some were distressed and anxious, albeit temporarily. Research protocols need to put in place safeguards where appropriate so that this group receives support and protection.

## Introduction

In the past few decades, worldwide, there has been an increase in research on interpersonal violence and trauma histories [1], [2]. With this increase, institutional review boards (IRBs) and researchers have raised ethical concerns about the studies [3], [4], in particular the potential negative impact (emotional reaction and distress) they may have on research participants [2], [5]. This concern has prompted some researchers to shift their attention towards empirically studying the impact of such research on participants [3], [4], [6].

At present, not much is known about how participants perceive being asked about interpersonal violence and trauma histories [7], [8], [9]. There has been little research on this area [1], [5]. Thus, distress and emotional harm of participants due to their participation in research remain a concern for all involved in research [8].

We have an obligation to both the field of research on violence against women, and in particular to the participants, to understand how being asked about their adverse experiences impact them [9]. Yet, the lack of data creates a major gap [10]. Very little is known about either adverse consequences or benefits derived by participants who have violence or trauma histories when participating in research that asks about such histories [4], [11].

Some authors argue that this leaves IRBs to make judgments about risks of research participation based on personal experiences, conjunctive assumptions and guesses, rather than on empirical evidence [4], [5], [7], [11], [12]. Researchers and IRBs have an important responsibility in ensuring that harm to research participants is minimized, while benefits are maximized [9], [11]. In order to carry out this task, researchers and IRBs need to, primarily, encourage and engage with research to better understand how participants themselves perceive risks and benefits in participating in research [5], [10], [11], [12]. Evidence from such studies can guide IRBs and researchers in making decisions about risk-benefit ratio of research proposals that aim to study interpersonal violence and other sensitive topics [3], [5], [7], [11], [12].

Whilst not much research has been done in this area, recent empirical evidence suggest that research participation for interpersonal violence and trauma survivors does not overwhelmingly distress participants, rather, participants report experiencing such research as beneficial [5], [6], [13]. This finding is consistent with findings from other studies which report that research participants, in particular those who have reported experiencing interpersonal violence and other traumas, seem to benefit from participating in research [1], [3], [6], [7].

This, however, does mean research participants do not get upset or distressed when asked sensitive questions or about their trauma histories [2], [12]. Yet, literature shows that a low percentage of participants report being distressed and or upset by research participation, and the negative effects, such as feeling distressed or upset, seem to be time limited and not overwhelming [2], [11]. Several studies report around 10% [3] of participants reporting some form of distress as a result of participation in research on interpersonal violence and other traumatic histories, but a few studies have reported higher percentages. For example, Johnson and Benight [6] enrolled 55 women (aged 18–65) currently recovering from domestic violence and recruited from domestic violence (DV) shelters, DV support groups, and other centers servicing abused women. They reported that 25% of participants reported being upset by research participation. Interpreting these statistics is complex as the distress of research participation may also be accompanied by a perception of benefit. Thus evidence suggests that most participants value being asked about violence and trauma histories in research and report that they would be willing to participate in such studies in future [2], [3], [5].

### Purpose of the Study

This study aimed to explore how participants perceived their experiences with a community-based survey of men and women (over 18 years) on prevalence of gender-based violence victimization and perpetration in the Gauteng province of South Africa. We wanted to understand participants’ perception on how the survey impacted them, how answering the survey questions had made them feel, and to establish whether they perceived the survey as distressing or helpful. We also wanted to understand if they had experienced any adverse consequences resulting from their participation in the survey.

The interviews were conducted 4 to 12 weeks (July-September 2010) after the main survey was administered. The survey questionnaires for men and women slightly differed in particular on phrasing questions on gender-based violence experiences. The questionnaire included items on socio-demographic characteristics, dimensions of adversity or trauma in childhood (emotional neglect and abuse, physical hardship and abuse; sexual abuse). There were questions on gender relations, control by the male partner in the relationship, sexual harassment, sexual relations and about witnessing domestic violence. Men were asked about the first time they ever raped, rape in the past year, whether they had ever raped a woman with peers, and attempted rape. Men were also asked about being victims of sexual coercion by other men. Women were asked about being victims of rape, relationship with the rape perpetrator, their age when it happened, where it happened, and whether the incident was reported to police. Men and women were asked questions on emotional, physical and sexual intimate partner violence perpetration (men) and victimization (women) [see 14].

### Setting

In the year 2010 a South African Non-Governmental Organization called GenderLinks (GL) collaborating with the South African Medical Research Council and the University of the Witwatersrand undertook a community-based survey to study the prevalence of gender-based violence in the Gauteng province of South Africa. The survey collected data in face to face interviews with a fieldworker using a structured questionnaire with women and men over the age 18 in 75 randomly sampled enumeration (EA’s) areas in the province.

For the qualitative study, from the 75 EA’s, we conveniently selected two EA’s that were closest to the South African Medical Research Council offices (place of work for both authors). Thus, the qualitative research was conducted in Soshanguve Township in the Gauteng Province, South Africa using multiple methods of data collection. Specifically, the qualitative study was conducted in the Thate Block and Siyakhula Extension (pseudonyms).

The Thate Block is predominantly a low-income area with few middle class families. Siyakhula Extension is relatively a new residential area which has originally been a squatter camp. It is mainly a poor area with some households being shack dwellings built of corrugated iron. These two sections (blocks) are approximately 4–6 kilometers apart.

Prior to conducting the qualitative in-depth interviews, YS (first author) had rented a room in the Thate Block and stayed fulltime for approximately 03 months (March to May in 2010) as an overt researcher. During this period he familiarized himself with the setting (both EA’s), collected general information on the community in order to be able to describe the context fully, mingled with the people and had unstructured conversations with the community members (not survey participants), learning as well their thoughts and feelings about research and their experiences of participating in research studies.

### Ethical Considerations

Ethics approval was provided by the ethics committees of the South African Medical Research Council and the University of the Witwatersrand. The purpose of the study, risks and benefits, informants’ rights, and the procedures involved in the study were explained to the informants. All informants signed an informed consent form. No incentive was given to the informants to participate in this research and we are not aware of any research adverse event having occurred during the period of data collection. In an attempt to ensure confidentiality and anonymity of the data presented in this article, names of all the informants have been changed, and the names presented in this article are all pseudonyms. We have also changed the names of the two EAs we conducted the study in. Furthermore, we are confident that the little description of the two EAs we provided above can not identify these EAs as Soshanguve Township is very large with many sections that are very similar in characteristics to the two EAs above.

## Materials and Methods

The article is based on 22 in-depth interviews, 12 conducted with men and 10 with women. The GL survey, to which this qualitative study was nested, randomly selected 20 households per EA for interview. One eligible men or female was systematically selected from those who slept four nights a week or more in the household and in total 511 women and 487 men participated in the survey [14]. The GL fieldworkers managed to interview 12 men in the Thathe Block and 12 women in Siyakhula Extension. Before the commencement of survey in these two EAs, YS requested the fieldworkers to invite the survey participants for the qualitative study and all 24 participants agreed to be contacted. They were initially contacted telephonically and thereafter met face to face for interviews. 11 men were interviewed by YS and 10 females were interviewed by a female researcher. Two females and one man could not be located for interview after several attempts. One man was interviewed twice after he requested another interview as he felt he had been dishonest in the first interview. (see Table 1 for informants’ background information). Interviews with men were conducted in isiZulu and those with women were a mixture of Zulu and seTswana. All interviews used a thematic guide and we audio-recorded the interviews. The guide for qualitative in-depth interviews with men was slightly different from that with women interviews. Informants were asked how the survey had impacted them, how answering the sensitive questions had made them feel, whether the research, directly or indirectly, was harmful or helpful to them and how, and whether they experienced adverse consequences as a result of their participation in the survey. In the qualitative in-depth interviews, informants were also asked to give life histories of violence, men were asked about violence perpetration and victimization and women victimization.

**Table 1:** Sketches of research participants.

Gender	Age	Relationship and health status	Social position	GBV experience
**Women**				
Mathapelo	34	Married	Not working	Forced sex by husband
Mapaseka	64	Single	Not working	Raped when young
Thandaza	50	Married	Not working	No
Busisiwe	38	Married	Not working	No
Cleopatra	62	Married	Not working	No
Nonhlahla	49	Widowed & HIV+	Not working	Abusive marriage
Mirriam	22	Dating	College	Abusive relationship
Margaret	46	Married	Not working	Abusive marriage
Nomusa	33	Single	Not working	No
Lebo	31	Dating	Not working	No
**Men**				
Thato	29	Dating	College	No
Papi	28	Dating	College	No
Mobutho	43	Dating	Not working	No
Vuyile	28	Cohabiting	College	No
Thato	26	Dating	Not working	Perpetrated IPV
Rorisang	29	Single	Selling cigarettes	No
Kelebogile	41	Dating & HIV+	Not working	Perpetrated IPV
Njabulo	43	Married	Not working	No
Oom-Dan	67	Married	Not working	No
Sipho	40	Cohabiting & HIV+	Social grant	No
Joe	45	Married	Working	Refused to answer

### Data Analysis

A grounded theory analysis was employed to analyze the data [15], [16], [17]. Data were analysed inductively. Initial analysis was performed by both authors separately and it included data from 23 in-depth interviews [17]. All interviews were digitally recorded. Audio-tapes were transcribed verbatim and translated to English by the first author and for the seTswana audio-tapes, we hired a seTswana speaking person to translate and transcribe the interviews. All transcripts were anonymysed and prepared for data analysis by the first author.

Initial codes generally corresponded with themes as set out in the interview guide. We went into the data and extracted relevant text and we grouped similar text under a theme that seemed to represent that particular text [17]. We then ran through the data identifying open codes. We did this by breaking the sentences into small segments identifying several codes within the same sentence [17]. At this early stage, we attempted to move up from the informants’ words and were abstract in labeling the codes [18]. We maintained consistency in labeling the codes so that it would be possible, at the end, to group similar codes together and produce categories [16]. At this stage, we came together and compared and discussed the codes until we agreed on which codes seemed to fit together to form categories [17]. We then followed the advice of Dahlgren et al. [16] and constructed concepts and the theory by finding axes between the codes and categories and thereafter identified the main category. We then explored what these data mean and interpreted them. In this last stage of the analysis, we compared the findings with the existing literature and made conclusions [16], [17], [19].

We present the findings by building a comparative argument through juxtaposing narratives of male and female informants, highlighting similarities and differences in their perceptions and experiences [17] of participating in the survey.

## Results

Many informants in this study reported to have appreciated the opportunity to participate in the survey. Some mentioned that the research afforded them an opportunity to talk about issues they don’t normally talk about. For example, Mapaseka (age 64) was raped when she was a teenager and got pregnant. She reported that at her home her grandmother and mother did not want to talk about her rape experience. As such she had kept it inside her and this affected her life tremendously. The survey interview provided her a rare opportunity to talk about the rape incident and this healed her somewhat. She explained:

Because as mothers, us mothers who are aged 64 we have met with many troubles in our lives. And you know when a person come from afar and she does not know you and she asked what are the things that you have experienced. I told her things and I felt pain as I was telling her and she was listening to what I was saying. My heart was sore but I told myself I have to talk about this, I have to talk about it so that it can come out of my soul (kumele ngiyikhulume ukuze iphume la emphefumlweni wami) because it caused me so much pain.

Other informants who had traumatic or life threatening experiences like Mapaseka, they too, reported that through the survey, they had an uncommon opportunity to talk. Nonhlanhla, a widow with five children who was HIV positive and had reported a history of being in abusive intimate relationships in her adult life, mentioned that she found the survey content to be relevant to her and saw it as an unusual opportunity to talk about her HIV status something she did not do often. A male informant Kelebogile, in his early 40’s, had a similar perception; he was HIV+ and reported that the survey interview had provided him a rare opportunity to talk about his HIV status in a space he perceived as safe.

### Attitudes, Perpetration, and Experiences of Gender-based Violence

The majority of women had an understanding that partner abuse comprised only physical and sexual abuse using physical force. For example, Mirriam reported that she had a boyfriend and perceived him as a good man who never gives her trouble. Yet when she was asked later in the interview “*How is your life with him?*”, she said: “*A lot of the time we fight, but not physically”.* Similarly, Mathapelo maintained in the interviews she had a non-abusive marriage, yet she later reported that her husband sometimes used non-aggressive methods of coercing her into sex, such as persistent pleading, subtle threats, accusations of infidelity and emotional blackmail; even though she had told him she was tired and did not want to have sex at the time. She explained:

…no, he does not force me with his hands (to have sex). He’ll say things like, “…just once…” things like that… The thing is he’s the type of person who wants something like it’s been forever and I don’t like being rushed and I don’t like being forced into something that I don’t want…for instance he sometimes come home and he wants to have sex and when you’re tired, you’re tired - he shouldn’t force you, shout at you, accuse you of sleeping around.

Other women reported in the interviews to be in abusive relationships or marriages or had had experienced partner abuse in their lives. Mapaseka had been raped when she was 19 years old by a man she knew from her community. Margaret reported that her husband often beat her. Nonhlanhla had also been in abusive relationships including in her marriage.

Seven men had fairly gender-equitable attitudes and views. In their narratives, they expressed disagreement with beating women, did not approve of it and expressed concern that it was very common in their community. Yet, Thabo a young man in mid 20’s clearly had gender inequitable views, attitudes and practices. In his interview he mentioned beating her girlfriend and felt justified beating her as she had cheated on him.

He said:

Uhm the thing is she had made me angry you see? She had made me angry and I beat her. But it was not that kind of beating as if I’m mad, I beat her up in a good way (ngamshaya kahle nje)…Uhm just slapping her, something like that. But I would not take a stone and beat her with it. I just slap her, you see? I’m just putting discipline in her (ngifaka icontrol kuphela) (laughing)… Ooh she was cheating, yes she was cheating.

### Concerns and Feelings About the Survey Process

In the interviews we asked the informants what their concerns and feelings were about the survey process; if there were any consequences, violence, distress and intimidation they experienced resulting from survey participation.

Data suggest that some women were left with fear post survey. They reported to have had fears that should their identities and information be disclosed, they may suffer violent reprisal from their partners.

In contrast men did not report this fear. Yet, they had felt that some questions were somewhat shocking to them, but not unusually invasive, and had understood why they were asked. Notwithstanding, five men reported that there were questions which had caused them conspicuous discomfort, although they had answered them. They viewed the questions as sensitive and personal. For them it was taboo to be asked about sex, condoms, HIV, intimate relationships and partner abuse. And some had feared negative ramifications that could potentially result from their disclosures. Our analysis reveals these men perceived such questions negatively because they were not used to being asked such questions.

Resulting from this discomfort, Thabo lied in the survey and reported that he had never beaten a partner whilst he had. He explained:

He asked whether “have I ever beaten a girl?” I told him “no” whilst I know that I have beaten a girl …eish I thought of many things, I thought of police, eish I really thought of many things (ngicabange izinto eziningi mfethu) my friend (laughing).

Other men reported that their discomfort was brought about by their fear of being judged or labeled negatively by the researcher because of their disclosures. For example, Kelebogile and Sipho reported discomfort in disclosing their HIV status in the survey, as such, the latter reported in our interviews to have been dishonest in answering the questions on HIV testing and status. Sipho explained:

Yes I did not tell him much, even with him I concealed a lot from him that I have AIDS; I don’t think I told him that. I did not tell him… I can’t really remember. But I think the thing that I did not tell him was that I have AIDS, no I did not tell him.

### Disclosure of Research Participation

A number of informants discussed their participation in the survey or were known by others (e.g. children, boyfriends, girlfriends, mothers and husbands and wives) to have participated in the survey. However, our data suggest that disclosure was done with fear by some women. Some women reported that they did not disclose much content of the survey; they had chosen to conceal particular information. It seems this was for different reasons. One informant Thandaza who described her marriage as non-abusive said that she did not see a need to tell her husband as the interview was about her. However, Margaret who reported to be in an abusive marriage and often beaten by her husband, reported that she did not disclose some particulars about the survey because she feared her husband would beat her. She explained:

I can tell him (my husband) but there are things I’ll tell him and other things that I won’t.

**Interviewer.** Why are there things that you won’t tell him?

Margaret: I couldn’t because he would hit me.

Mirriam, a young unmarried woman currently in an abusive relationship, told her boyfriend about the full content of the interview and she felt threatened by the remarks he made. She posited:

I only told him that…that day when they did the interview, he asked me why they asked me if he’d ever hit me, did I want them or what, and I said don’t talk like that. He asked whether they wanted people to get kicked out of their homes or what… I felt bad when he said do I want [for a sexual/intimate relationship] those people… I felt bad because he’s not supposed to speak that way, he should have just said okay.

Nonhlanhla, a widow, who had been in abusive marriage and relationships in the past, but did not describe the present relationship as abusive, stated that she did not inform her new boyfriend that she was asked about rape because it was not important for him to know. Mathapelo and Busisiwe reported that they discussed everything they were asked in the survey with their husbands without negative reaction from them. Both women had reported that their husbands were not physically abusive.

Most men did not discuss their survey participation with anyone, yet giving reasons that differed from those of women. Young men like Thabo, Rorisang and Thato who stay only with their mothers stated that they did not feel comfortable to discuss some survey questions with their mothers. Rorisang who reported to be addicted to nyaope- a cocktail of dagga and cheap heroin- which is very popular in this setting mentioned that he did not discuss his survey experience with his friends as they undermine him and don’t take him seriously. Also, he did not have the kind of relationship with his mother that would allow him to talk about personal issues.

However, other men reported to have discussed their participation in the survey with their mothers, wives, friends, and girlfriends. These men said they had a special relationship with the people they told and trusted them, so they felt comfortable to talk about the content of the survey with them.

Men reported positive reactions from the people they told about their survey participation. For example, Kelebogile’s mother was happy that he had participated in the survey and was particularly keen to know if he had reported that he was HIV positive. She was pleased to learn he had. In contrast, Vuyile’s girlfriend was not bothered by his participation in the study, yet she was unhappy that he had reported about their private life.

### Impact of Research on Participants

Mapaseka did not experience overwhelming and prolonged distress resulting from the survey questions, even though she had spoken about her rape: She explained.

what I can say is that I feel very happy. I don’t have regrets in anyway, my spirit is at ease, (ngizizwa ngikhululeke kabe, angisoli ndawo, kushukuthi umoya wami umnandi kabi), maybe with time, it will heal completely in my heart and in my spirit. Maybe it will heal completely and no longer think about it (rape incident)… It is better to speak than keeping quiet about a matter.

From this narrative, it is apparent that speaking about the rape incident caused Mapaseka pain, yet she attached value in talking and had perceived it cathartic.

Similarly, for Nonhlanhla the survey had made her to think about her husband’s death, and this caused her pain at that time. She was HIV positive and had suspected that her husband died of AIDS related illness, but he had not told her he had AIDS. She explained:

I spoke to her but I felt that pain, because it reminded me of something I had forgotten that happened a long time ago…they [questions] were not hard to answer because they are things of the past but it was hard talking about his death but otherwise the talking about being HIV positive didn’t bother me at all because I know which stage I am in.

Mathapelo mentioned that the interview caused her to think about the abuse she witnessed when she was a child, where her uncle was physically and emotionally abusing her aunt, and reflecting on this had made her to feel sad.

Similarly, some men reported that some survey questions had made them reflect on painful experiences about their lives. For example, Sipho and Kelebogile mentioned that the survey had made them to think about their health condition, that they were HIV positive, something they prefer not doing. Thabo who had reported to be physically abusive to his girlfriend reported that the questions about partner abuse had made him to reflect on his own actions of beating his partner, and had a realization that he had abused her. As well, Rorisang mentioned that the survey made him to think about his drug addiction problem and he felt sad being reminded it was harmful to his health.

Our data suggest that women like Mapaseka, Cleopatra and Nonhlanhla who had reported to have experienced relatively major adversities in their lives, [rape, death of a loved one, and HIV], the survey made them to relive those painful experiences causing them sadness and pain at the time.

In the interviews informants were asked how the survey had impacted them. Although some informants had mentioned that talking about some experiences caused them sadness and pain, they felt the pain was temporary and not overwhelming. Furthermore most informants mentioned that the interview itself provided catharsis for them in different ways. It seems informants appreciated the opportunity to speak freely about the problems they have been bottling inside; a safe environment like the one seemingly provided by the survey interview, allowed them space to do this.

For some women, the experience of participating in the survey and the information they derived from the survey, had an empowering effect on them. For example, Mathapelo reported that after the survey she tried to communicate her displeasure to her husband about him *forcing her to have sex when she is unwilling.*

We found the same for men. Many said the survey was somewhat educational and empowering as it made them to reflect on important aspects of their lives, in particular implications of their behaviours, something they don’t normally do.

### On the Referral Support System

In the interviews we asked the informants: did thinking about the issues that were asked in the survey cause you any distress? If yes, we asked: what kind of support they felt they needed.

Three informants (two women and a man) did not recall being given a list of referral support services they could go to by the field workers. However, many women, including those who reported to have had experienced partner violence or were in abusive relationships, reported having needed support for non-violence or study related issues. For example, Thandaza had needed assistance for the arthritis she was suffering from. She also mentioned that she needed help with the financial challenges at her home and being assisted with organizing a grant as she was ill.

Mapaseka said she needed help with claiming maintenance from the man who raped and impregnated her. It was evident that whilst Mapaseka had reported to have been emotionally and psychologically affected by her rape experience, the interview itself did not cause her overwhelming distress that may have warranted professional intervention. It may be that she had healed over the years. Her narrative supports this interpretation:

yes they gave me the paper (list of local referral services) but I have not looked at it properly…there was no help I needed for the things the researcher asked me about.

Nonhlanhla, who had reported that the interview had caused her to think about the death of her husband, said she would have been happy if the researchers had offered her a job and help with her municipal debt. Mirriam, who reported being in an abusive relationship, mentioned that she did not know the kind of support she needed because of the survey questions. This is congruent with what Margaret said. She had reported to be in an abusive marriage in which her husband beats her. Yet she said she did not need support resulting from answering survey questions. Likewise, although Mathapelo had said she often felt her husband forces her to have sex with him, and herself had equated this to rape, when asked the same question she posited:

no there isn’t help I needed because of the things I was asked in the survey…I’ve been alright after the interview; because I was able to explain what happened to someone else.

Almost all men in the study said they did not need any support because of the questions they were asked in the survey. Therefore, we asked them to think hypothetically if they had been affected negatively by the survey questions, what form of support they would have needed. Almost all reported that talking to significant people in their lives was their first preference. Mobutho’s narrative is illustrative:

Well I think the main support is still to talk to family members around. I think they are the ones who can support you all the way with that problem and comfort you. They are the ones who can comfort you when experiencing that thing; that is my belief; only family members can help you.

He further said:

counseling is better, counseling is one of the cures that can heal those wounds. I support even counseling, but my first preference is to talk to family members. Then if you are not happy with their support, then you can take plan B and go for counseling. But my first preference is family members and plan B is counseling.

Rorisang was an exception here as he felt if he had been distressed he would have sought comfort from smoking nyaope as he had no one to speak to. Sipho and Kelebogile, who were both HIV positive, however felt they would have needed support related to their ill-health and financial assistance.

## Discussion

Our findings suggest that some women remained with fear after the completion of the survey. From these women narratives, it was apparent that they were worried about the potential physical harm that could result as retaliation, mainly from their partners, if there could be a breach of confidentiality. Our analysis shows that mostly these women had a history of partner violence or other forms of GBV. The only excerption here was Busisiwe who reported not experiencing abuse from her marriage. Despite not experiencing physical abuse in her marriage, she was worried that her husband would react violently if he discovered she discussed their “private” information in the survey.

In contrast, no man reported fearing physical retaliation from a partner. This, perhaps, is unsurprising considering the patriarchal nature of the South African setting where men mostly have control and dominance over women and often perpetrates violence against women [20]. This may explain why only female informants reported fearing possible retaliation from their partners.

Many men in this study reported to have been shocked by the type of questions they were asked in the survey. They found some survey questions too personal and sensitive (e.g. questions on sex, number of sexual partners, HIV and partner abuse), and this caused discomfort for them. Our analysis reveals that the few men who reported emotional reaction to these questions, had also reported perpetrating intimate partner violence or were HIV positive, and thus, may have been uncomfortable to talk about these issues as that either reminded them of and invited them to confront and evaluate their own actions [21] and, for the others, illnesses.

Some participants, like Thabo and Sipho, mentioned in the qualitative interviews that they did not report honestly in the survey about perpetrating partner abuse or their health status (in particular HIV) but were candid about these in the qualitative interviews. The reason for this difference may be that YS had resided in the community for three months prior to conducting the interviews with men, and a sense of trust and confidence in the interview may have had developed potentially creating space for participants to answer questions more honestly. The one-off nature of the survey may have limited the space for a rapport to be established between participants and researchers and that, for some participants, may have led to discomfort in reporting sensitive and personal information.

Our data suggests that whilst a number of informants had emotional reaction to some survey questions, the vast majority thought the survey had a positive effect on them. This is similar to a finding reported by Griffin et al. [13] that whilst participants in their study had recently suffered acute sexual and domestic abuse and were subjected to extensive psychological and physiological assessments there was a high level of interest in the study with low levels of distress to assessment procedure.

Whilst many authors have studied the perceptions of or risks of research participation in interpersonal violence or trauma survivors, their focus has mainly been on emotional reaction or psychological risks [3], [4], [10], [13], with lack of focus on risk for physical harm to participants. Women research participants have been viewed as a vulnerable group and that, often, may be exposed to, as Wasunna [22] argued, immediate or perpetual danger of abuse through their participation in research [23], [24], [25], [26].

In an effort to protect research participants, (especially women) from potential abuse, researchers often do not introduce their studies as that on GBV at community level, and only reveal the actual focus of the research to the selected women only [23], [25]. Additionally, researchers often advise the participants to not divulge the focus of the research to others, explaining that this is done to maximize participant protection [25]. However, IRBs and others have raised concerns that this may be construed as deception, and view this safeguard as ethically questionable. Jewkes and Wagman [26] have, however, argued that in the South African setting, community gatekeepers are often men, whom themselves could be perpetrators of GBV and may hold such views that legitimate dominance and control of women by men. Therefore they argue that under these circumstances, this ‘form’ of deception on community gatekeepers is justified; both in terms of concealing the true focus of the research and in terms of concealing the identity of individual research participants.

In keeping with Jewkes and Wagman [26], we support a view that this form of deception should be for community gatekeepers, and not the participants. The survey was broadly termed and had included many other questions that were not GBV related (e.g. income, abortion, schooling, food etc), yet in the qualitative interviews, informants generally understood the focus of the research as being on issues of gender, sexuality, women abuse, gender relations, which all fall in the realm of GBV.

Whilst some informants, may have had heeded the advice not to tell others about the focus of the survey, the vast majority reported to have discussed their research participation, with some disclosing the full content of the survey. Therefore, in the interviews, we probed informants in order to understand whether this placed them at risk of physical harm or other form of abuse by third parties.

In terms of perceived risks of disclosing research participation and content we found gender differences. All men reported no negative reaction, in particular, from their wives or girlfriends. The same reason we gave about control and dominance of men over women in this setting should explain this phenomenon. In contrast, although not for all women, our data suggest that some women perceived risk in disclosing the full content of the survey, and indeed some received negative responses from their intimate partners, that were somewhat threatening. One woman [Margaret] who reported in her interview to be in an abusive marriage, stated that she did not disclose the survey content because she feared being physically assaulted by her husband. We also think she may have also heeded the advice from the fieldworker not to disclose the survey content.

Among women who had disclosed the full content of the survey, we noted differences according to interpersonal violence histories. Women who were in abusive relationships reported negative reactions that were relatively threatening from their partners. In contrast, women who had reported no abuse in their relationships reported that their partners were not bothered by the survey content. Whilst no woman reported being physically assaulted by an intimate partner because of participating in a GBV survey, this finding suggests that some women may be put at risk of harm if the content of the GBV survey is known by violent and controlling men [22]. Jewkes and Wagman [26] argue that violent men may be offended upon knowing that his partner had discussed his violent behavior in the study, and thus react by physically assaulting her as a form of punishment.

Our findings support the WHO [27] recommendation that the actual focus of GBV survey should be concealed at community level, told only to participating women, and that women participants should be advised not to disclose the focus of GBV in the survey [see also 28]. This recommendation protects a particularly vulnerable subgroup of women i.e. those in abusive or potentially abusive relationships. Our data reveal that full disclosure of GBV focus of survey to abusive and controlling men, may trigger violence, and lead to harm for women participants. This aspect of risk to research participants is of particular importance in our understanding of risks to research participants. Our study provides important evidence on this risk; however, more research is needed, from this setting and elsewhere, in order to adequately understand the characteristics of participants who are more vulnerable to physical harm and the circumstances under which this harm could occur. This can maximize participants’ protection.

IRBs and researchers have raised concern that interpersonal violence and trauma survivors as research participants may be emotionally or psychologically harmed by being asked about their adversarial histories [2], [11], [13]. This concern is, however, based on anecdotal evidence, or often, assumptions and worst case scenarios of research atrocities [11], [13]. Our study findings reveal that although there was no remarkable difference between men and women in reporting distress resulting from research participation, slightly more women reported sadness or pain when reflecting on painful experiences, than males. This finding is analogous to that reported by Kuyper et al. [11] in their study with young people in the Netherlands. They reported that women expressed more distress because of the questions asked as compared to men.

While in their study DePrince and Freyd [4] did not find evidence that cultural taboo may be the cause of upset for survivors of abuse and interpersonal violence, in the present study some men felt it was unusual to be asked some of the things in the survey, as such, they were somewhat upset by this. However, we also think some men may have been upset with the partner abuse questions because they perceived such questions as somewhat incriminating [10], [21], and for others, questions on HIV status [Sipho and Kelebogile] and drug abuse [Rorisang] may have made them to reflect on their actions and to think they were to blame for their current conditions.

Authors have argued that the ‘mere presence of sexual abuse history does not predict women’s negative emotional reactions to research, but that assault characteristics and post assault attributions and distress levels also play a role’ [1]. Griffin and colleagues [13] concur, they reported that while women in their study had recently suffered acute sexual and domestic abuse and were subjected to extensive psychological and psychophysiological assessments, they did not get damaging effects from this experience. Similarly, Johnson and Benight [6] found that the recent domestic violence victims tolerate trauma research fairly well. In the present study, although some informants had reported about traumas that had happened years ago, some were still in abusive relationships and others had HIV or had AIDS, yet they did not find it emotionally damaging to talk about such experiences in the survey. In support of this reasoning, Johnson and Benight [6] argue that ‘the ability to tolerate research that asks about sensitive and traumatic experiences may be related to coping self efficacy, the perceived ability to cope with recovery demands.’

Our data suggest that the emotional reaction to survey questions, to those who reported it, was temporal and not overwhelming, and thus would not be categorized as emotionally or psychologically harmful [4]. Jorm et al. [3] did a systematic review of literature investigating whether there is evidence that participation in psychiatric research causes harm. Particularly focusing on long-term effects of research participation, these authors concluded that there appears to be little evidence to show any long-term harm to participants even if research studies traumatic experiences. In the current study, not a single informant, reported effects of survey questions that suggested that the impact would have warranted intervention. Kuyper et al. [11] argue that emotional effects resulting from research participation may quickly fade away, and this may explain why our informants, even though had reported distress, also stated that they did not feel they needed any help. We argue that the distinction between sadness and pain and being psychologically damaged in the research context is important to make as the former seems not to equate the latter, as often assumed.

Our data shows that whilst a number of informants had felt discomfort with some survey questions, none regretted participating in the survey. Rather, including those who had reported distress, an overwhelming majority reported positive feelings about the survey [3]; with a number of informants mentioning that the survey interview itself had provided catharsis for them. In Edwards et al. [1] study, women who had experienced child sexual abuse and those who experienced adult sexual abuse reported more personal benefits to research participation as compared to women without abuse histories. Similarly, although with a somewhat younger sample, Kuyper and associates [11] enrolled 889 sexually experienced young people in the Netherlands examining the effects of asking the participants about various sexual topics in a large-scale sexuality study. They found that the overwhelming majority of participants reported positive feelings and benefits from research participation [11].

In the current study, a number of informants, in particular those who had major adversities in their lives (e.g. sexual assault, IPV, HIV), mentioned that they do not often get a safe space to talk about their traumatic experiences, and for them, the survey had provided this. As such, they found research participation cathartic as it allowed them space to relate their experiences to a person who was willing to listen and empathetic. This finding is consistent with Johnson and Benight [6] view that research participation may serve as a catharsis and or a motivation to seek help. Additionally, Campbell [29] in her book about the impact of researching rape argues that the ‘very act of research participation is something of an intervention in its own right.’ Our data provide support to this notion. In a setting like South Africa where women often do not have a “voice”, our findings show that women in this study felt acknowledged by being given a safe space to voice out their inner and commonly suppressed feelings.

In 2001 the WHO published the Ethical and Safety Recommendations for Research on Domestic Violence Against Women guidelines. Reflected in these guidelines is also a recommendation that ‘field researchers should be trained to refer women requesting assistance to available local services and sources of support. Where few resources exist, it may be necessary for the study to create short-term support mechanisms’. This recommendation provides a duty for GBV researchers, but does not clearly articulate the boundaries of such a duty thus opening it to various interpretations [22]. The dominant interpretation has been that for GBV research with women to meet the ethical requirements, it has to make a provision for referral to local services [22], [26]. As such, studies on interpersonal violence often employ varying safeguards that include offering to provide referrals to local counseling services [9]. This has been the case even though there has been little or no empirical evidence suggesting it is a needed and useful safeguard in this field [26].

Adhering to this recommendation, the survey had made a provision for referral to local services for all participants in the survey [14]. The setting of the survey is well resourced thus services were readily available; and therefore not necessary to create short-term mechanisms. In the present study we explored whether the participants perceived the emotional reaction they had to the survey questions warranted professional intervention, and which participants needed this. We had anticipated that those who reported major adversities in their lives would be more likely to report needing help after the survey, yet none of the informants reported having needed support because of the survey questions. This is consistent with the findings from a study in Netherlands where Kuyper et al. [11] reported that of the 889 participants, one in four reported distress (like feeling down or sad), yet only 3.5% of the sample experienced a need for help.

In the current study we found no difference according to interpersonal violence or trauma experiences or gender in reporting the need for help. However, some informants reported that had they felt they needed emotional support because of the survey questions, they would have preferred to talk to family members rather than attending professional counseling. They perceived that family members knew them better and would thus provide better support.

Much of the published research on this area is from North America and Europe and we are not aware of any from South Africa. Therefore data from the current study is important as it provides evidence for risks and benefits perceived by research participants from a South African perspective. This will aid, as well, South African IRBs and researchers in their decision making about the risk-benefit ratio of studies on interpersonal violence and trauma in South Africa and similar settings.

This qualitative study was conducted one to three months after the survey; therefore it could not capture participants’ long-term reactions to and consequences of survey participation. As discussed above, some women had remained with fear (of violent reprisals) after participating in the survey. Yet during the period between one to three months post survey, in the qualitative interviews, none reported these fears being realized. Specifically, none reported being physical harmed as a punishment for research participation.

Studies that require people to recall and report about past events, especially feelings and emotions, after some time had passed, may have a problem of recall bias. In the current study, few informants could not recall survey questions that distressed or upset them. We argue that, had the experiences been harmful with long-lasting effects, informants would still be experiencing the effects and thus able to report those in the interviews.

Whilst the participants in this qualitative study had initially been randomly selected to participate in the survey [14], it is the nature of qualitative research that the findings are not generalisable. Their importance is that they are the lived experiences of survey participants and may thus be important to guide researchers on how to approach community-based studies involving human participants in this and similar settings elsewhere [16].

### Conclusion

We have presented findings showing that the majority of participants in this study, including those who had endured violence, did not feel answering the survey questions had caused them emotional or physical harm. Some had reported feeling sad and upset on reflecting on painful life experiences during the survey interview, but they felt these emotions quickly went away, and most of them perceived participating in the survey positively. However, we suggest that even in the light of evidence that some participants were temporarily distressed and had been anxious about menacing responses from their partners when they told them about survey participation, research protocols need to put in place safeguards. As such we recommend that future community-based research should adhere to the WHO guidelines and safety recommendations [27] including concealing the violence focus of the research and to continuously advise women participants not to disclose the focus of the research to third parties, in particular their partners. We suggest that this should be practice in all community-based research involving women as it is currently not well understood which men may react violently and what may specifically make them to react violently.
